# The Role of the Complement System in the Pathogenesis of Infectious Forms of Hemolytic Uremic Syndrome

**DOI:** 10.3390/biom14010039

**Published:** 2023-12-27

**Authors:** Piotr P. Avdonin, Maria S. Blinova, Galina A. Generalova, Khadizha M. Emirova, Pavel V. Avdonin

**Affiliations:** 1 Koltzov Institute of Developmental Biology RAS, ul. Vavilova, 26, 119334 Moscow, Russia; maria.s.blinova@gmail.com (M.S.B.); pvavdonin@yandex.ru (P.V.A.); 2 Saint Vladimir Moscow City Children’s Clinical Hospital, 107014 Moscow, Russia; gangen@yandex.ru (G.A.G.); kh.emirova@outlook.com (K.M.E.); 3 Department of Pediatrics, A.I. Evdokimov Moscow State University of Medicine and Dentistry, 127473 Moscow, Russia

**Keywords:** hemolytic uremic syndrome, complement system, thrombotic microangiopathy, eculizumab, *Escherichia coli*, Shiga toxin, STEC-HUS, hemolytic anemia, thrombocytopenia, acute renal failure, pathogenesis, endothelium

## Abstract

Hemolytic uremic syndrome (HUS) is an acute disease and the most common cause of childhood acute renal failure. HUS is characterized by a triad of symptoms: microangiopathic hemolytic anemia, thrombocytopenia, and acute kidney injury. In most of the cases, HUS occurs as a result of infection caused by Shiga toxin-producing microbes: hemorrhagic *Escherichia coli* and *Shigella dysenteriae* type 1. They account for up to 90% of all cases of HUS. The remaining 10% of cases grouped under the general term atypical HUS represent a heterogeneous group of diseases with similar clinical signs. Emerging evidence suggests that in addition to *E. coli* and *S. dysenteriae* type 1, a variety of bacterial and viral infections can cause the development of HUS. In particular, infectious diseases act as the main cause of aHUS recurrence. The pathogenesis of most cases of atypical HUS is based on congenital or acquired defects of complement system. This review presents summarized data from recent studies, suggesting that complement dysregulation is a key pathogenetic factor in various types of infection-induced HUS. Separate links in the complement system are considered, the damage of which during bacterial and viral infections can lead to complement hyperactivation following by microvascular endothelial injury and development of acute renal failure.

## 1. Introduction

Hemolytic uremic syndrome (HUS) is a form of thrombotic microangiopathy (TMA), which is characterized by the presence of three pronounced symptoms: thrombocytopenia, acute renal failure, and microangiopathic hemolytic anemia. This syndrome is one of the most common causes of renal failure in children. The development of HUS is based on a whole range of different causes that determine the course of the disease, treatment approaches, and outcome. These may be infectious diseases, cobalamin C defects, mutations in the gene encoding diacylglycerol kinase ε (DGKE), genes of complement system factors, antibodies to complement factor H, organ and tissue transplantation, tumor, autoimmune diseases, etc. The etiology of HUS lay down the basis of its classification. Initially, it was accepted to divide all cases of HUS into two main groups: typical and atypical HUS. Typical HUS usually includes all cases caused by infection with hemorrhagic strains of *Escherichia coli* and *Shigella dysenteriae*. In this case, the key pathogenic factor causing the development of HUS are the toxins produced by pathogenic bacteria. At the same time, all cases not associated with infection with strains of *E. coli* and *S. dysenteriae* were traditionally classified as aHUS. As data on the causes and mechanisms of HUS development have accumulated, the classification of HUS has changed [1,2,3]. In 2016, an international expert group of clinicians and basic scientists studying HUS proposed a classification dividing all cases of HUS into seven groups [4]:

- HUS caused by hemorrhagic Shiga toxin-producing *E. coli* (STEC-HUS);
- Secondary HUS (due to cancer, organ and tissue transplantation, medications, autoimmune disorders, malignant hypertension, and HIV infection);
- HUS associated with infections caused by the H1N1 influenza virus and *S. pneumoniae*;
- HUS associated with cobalamin C defect;
- HUS associated with mutations in the DGKE gene;
- HUS caused by dysregulation of the alternative complement pathway (mutations in complement genes and antibodies to factor H);
- HUS of unknown etiology.

This classification is based on etiological features, but a deeper understanding of the pathogenetic mechanisms of HUS is also necessary to develop rational treatment methods. The results of recent studies indicate that the complement system is involved in the pathogenesis of HUS in these infections. In some cases, it has been established that the development of pathogen-induced HUS is accompanied by activation of the alternative pathway of the complement system. On the other hand, in the case of HUS that is associated with disturbances in the regulation of the alternative complement pathway, the question remains open as to what events serve as a trigger for its development in these cases. The presence of mutations in the genes of complement factors does not in itself trigger the pathological process. Since the complement system is part of the human immune system, it is logical to assume that infectious diseases can act as a trigger leading to a disruption of its functioning. This is confirmed by statistical data according to which in 79% of cases, a relapse of aHUS develops against the background of infectious diseases, mainly viral [4]. It is important to note that the range of infectious diseases that can provoke the development of HUS is expanding. In this review, we tried to collect information about all currently known infectious agents that can cause HUS, and the role of the complement system in the pathogenesis of HUS in infectious diseases. Since the complement system is in close relationship with the blood coagulation system and the result of the development of HUS is thrombosis, we tried to pay special attention to the interaction of these systems.

## 2. Complement System

The role of dysregulation of the alternative complement pathway in endothelial cell damage and the development of TMA was first considered in 1998, when abnormalities in the CFH gene, encoding complement factor H, were discovered in patients with HUS [5]. Normally, the complement system plays an important role in the body’s humoral defenses, enabling pathogen detection and elimination [6]. The complement system consists of more than 40 proteins, including regulatory proteins and complement receptors [7]. Complement factors are predominantly synthesized by hepatocytes and are present in the blood plasma in an inactive form. Synthesis of complement system components can also occur in neutrophils (C7) [8] and adipose tissue (factor D) and, to a lesser extent, in macrophages/monocytes, endothelial cells, keratinocytes, and renal epithelial cells [9]. Data also appeared on the existence of a local complement system and the presence of proteins and complement receptors inside immune and non-immune cells. This system was called the complosome [10]. The functions of the complement system are extensive and are not limited to protecting the body from pathogens, as originally thought.

Complement factors are involved in the opsonization and lysis of pathogens [11,12], recruitment of phagocytes for their destruction [13], modulation of smooth muscle contraction and vascular permeability, removal of immune complexes and cellular debris [14], angiogenesis, tissue regeneration and wound healing, provision of proliferative signals for adaptive immune cells [15], initiation and enhancement of the adaptive immune response [16], and neuroprotection [17,18].

**Figure 1:**
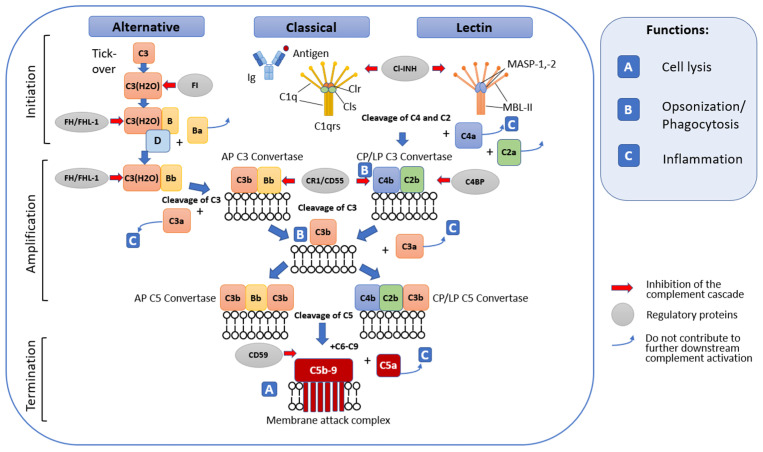
Complement activation pathways. The CP and LP are activated via antibody–antigen complexes or by sugar moieties on the surfaces of bacteria, respectively, whereupon C4b is surface deposited in a complex with C2b, forming the LP/CP C3 convertase (C4bC2b). The AP is constitutively activated by spontaneous thioester hydrolysis. Either the LP/CP or AP C3 convertase (C3bBb) may result in deposition of surface C3b [B] and generation of respective C5 convertases. C5b production triggers the assembly of the lytic membrane attack complex [A] by the addition of C6, C7, C8, and multiple C9 molecules. C3a and C5a, the smaller fragments, are referred to as anaphylatoxins. They mediate chemotaxis, inflammation and do not contribute to further downstream complement activation [C]. Under physiological conditions, complement activation is tightly controlled by the regulators of complement activation (FI, FH/FHL-1, CR1, CD59, C4BP, CD55, CI-INH).

Complement activation occurs through one or several pathways, which are called the classical, lectin, or alternative pathway (Figure 1). 

The end result of complement activation is the formation of the membrane attack complex, which creates pores in the cell membranes of some pathogens and infected cells, which can lead to their death.

All components of the classical complement pathway and membrane attack complex are designated by the letter C followed by a number. Native components have a simple numerical designation, for example, C1 and C2. The numbering does not reflect their place in the chain of molecular interactions during complement activation, but the order of discovery of the complement factor. In this regard, the sequence of reactions C1, C4, C2, C3, C5, C6, C7, C8, C9 does not look entirely logical. Activation of the complement system is accompanied by the cleavage of native factors with the formation of complexes with specific activities. The products of cleavage reactions are indicated by adding lowercase letters. The larger fragment is designated by the letter b, and the smaller fragment by a. The exception is C2; the larger active cleavage fragment has long been designated C2a. Instead of being numbered, the components of the alternative pathway are designated by different capital letters, for example, factor B and factor D. As with the classical pathway, their cleavage products are designated by adding lowercase letters a and b; thus the large fragment B is called Bb, and the small fragment Ba. Finally, in the lectin mannose binding pathway, the first enzymes activated are the mannan-binding lectin-a-associated serine proteases MASP-1 and MASP-2.

The classical pathway plays a role in both innate and adaptive immunity. It is initiated upon recognition of antibody–antigen complexes or surface-bound pentraxins by complement fragment C1q. C1q is part of the C1 complex, which consists of one molecule of C1q associated with two molecules of each of the zymogens C1r and C1s. Its binding to the target causes a conformational change in the C1r2–C1s2 complex, which leads to the activation of autocatalytic enzymatic activity in C1r; the active form of C1r then cleaves the associated C1s to form an active serine protease. Once activated, C1s cleaves C4 and C2 to produce two large fragments, C4b and C2b, which together form the classical pathway C3 convertase (C4b2b), and two small fragments, C4a and C2a, whose functions are not completely clear. In turn, C3 convertase, remaining on the surface of the pathogen, cleaves a large amount of C3 to form fragments C3a and C3b [19]. Fragment C3a is an anaphylatoxin with proinflammatory activity. The C3b fragment either covalently binds to neighboring molecules on the surface of the pathogen, allowing recognition and phagocytosis by phagocytes, or binds to the C3 convertase to form the C5 convertase C4b2b3b. Unbound C3b is inactivated by hydrolysis.

The lectin pathway is triggered by the recognition of microbial glycans by pattern recognition receptors (PRRs). These include (1) mannose binding lectin (MBL) of the collectin family and (2) ficolins. MBL-associated serine proteases (MASP-1 and MASP-2) are evolutionarily related to C1r and C1s and function in a similar manner [20]. When interacting with MBL, MASP proenzyme molecules are activated and acquire the ability to cleave complement components C4 and C2, similarly to the C1 complex. Further reactions of the lectin and classical pathways coincide.

The alternative pathway is associated with constant spontaneous hydrolysis of the thioester bond in the C3 molecule. The resulting C3(H_2_O) molecule interacts with factor B. The binding of factor B to C3(H_2_O) allows a plasma protease, called factor D, to break down factor B into Ba and Bb. The Bb fragment remains associated with C3(H_2_O) to form the C3(H_2_O)Bb complex. This complex is a liquid phase C3 convertase, and although it is only produced in small quantities, it can cleave many C3 molecules into C3a and C3b. The amplification phase begins, as a result of which the cleavage of factors B and C3 increases. The accumulating fragment of C3b, having contacted the surface of the host cell or pathogen, is able to bind factor B, ensuring its cleavage by factor D with the formation of the alternative pathway C3 convertase, C3bBb. C3bBb activity is stabilized by factor P or properdin, which is found on cell surfaces with reduced sialic acid content (e.g., foreign cell membranes). In turn, the binding of C3b to C3bBb results in the formation of the C5 convertase C3bBbC3b [19].

### 2.1. General Terminal Stage of Complement Activation

The terminal stage of the complement activation cascade is triggered by the formation of C5 convertase. C5 convertases of the alternative and classical pathways act in a similar way. They split C5 into C5b and C5a. In this case, C5a plays the role of a chemotactic and anaphylactogenic molecule, while C5b, having contacted other complement components fixed on the cell membrane, participates in the formation of the lytic membrane attack complex (MAC) [21]. In addition to pore formation and cell lysis (especially Gram-negative bacteria), the functions of MAC also include stimulatory activity in the polarization of T-helper cells and the role of soluble MAC in platelet activation [22,23].

### 2.2. Complement Regulatory Mechanisms

Excessive activation and dysregulation of complement and misrecognition of cellular debris or grafts can lead to various pathological conditions [21]. That is why restraining regulatory mechanisms acting at different stages of the cascade reaction are necessary. Regulatory factors can be present both in the liquid phase and on the cell surface. The major regulators of fluid phase complement include serum C1INH, C4BP, protein S, factor H, factor I, and anaphylatoxin inhibitor AI. Cell-associated regulators include CD55 (DAF), CD59 (MAC-IP or Protectin), MCP (or CD46), CR1 (or CD35), and CRIg [19]. Complement regulators act in different ways (Figure 1). For example, C1INH inactivates the C1r and C1s proteases in the C1 complex of the classical complement pathway; CD55(DAF) causes degradation of C4b2b and thus interrupts the formation of classical and lectin pathway C3 convertase, and CD59 prevents final assembly of the membrane attack complex [6,19].

A major role in the inactivation of C3bBb is played by complement factor H (CFH), a plasma glycoprotein consisting of 20 short consensus repeats (SCRs). Factor H is able to bind C3b, thereby preventing the cleavage of C5 and factor B on cell surfaces and inhibiting the formation of C3 and C5 convertases. In addition, factor H is a cofactor for factor I, a regulator that mediates the proteolytic cleavage of C3b [19]. Factor H acts both in the fluid phase and on cell surfaces by recognizing host cells directly through specific glycosaminoglycans and sialic acid or indirectly, for example through C-reactive protein (CRP) [24].

The proteins Vitronectin and Clusterin also take part in the regulation of terminal MAC assembly. Vitronectin, also known as protein S, preferentially binds to C5b-7 and interacts with C9, inhibiting its polymerization, thereby preventing the formation of a lytic pore in the membrane of the attacked cell [25]. Clusterin specifically binds to C7, the beta subunit of C8 and C9, also inhibiting the polymerization of C9 [26]. 

The complement system is also regulated by other systems, including the blood coagulation system, which we will discuss below.

## 3. Interactions of the Complement System with the Blood Coagulation System

The complement system and the blood coagulation system have a common evolutionary origin, which led to the presence of common activators and inhibitors and synergy in their work, which is extremely important for the body’s fight against pathogens [27]. The function of the blood coagulation system is not only to ensure the integrity of the cardiovascular system, but also to localize the source of infection if it enters the body and stimulate inflammation mediated by the complement system. In turn, the complement system engages the blood coagulation system in the fight against pathogens. A striking example of such interaction is the syndrome of disseminated intravascular coagulation in sepsis. Thus, the functional relationship between the complement system and the blood coagulation system provides protection for the body. Dysregulation of the activity of these systems and the pathways of interaction between them can lead to the development of severe complications, including complement-associated thrombotic microangiopathies.

### 3.1. Blood Coagulation System

**Figure 2:**
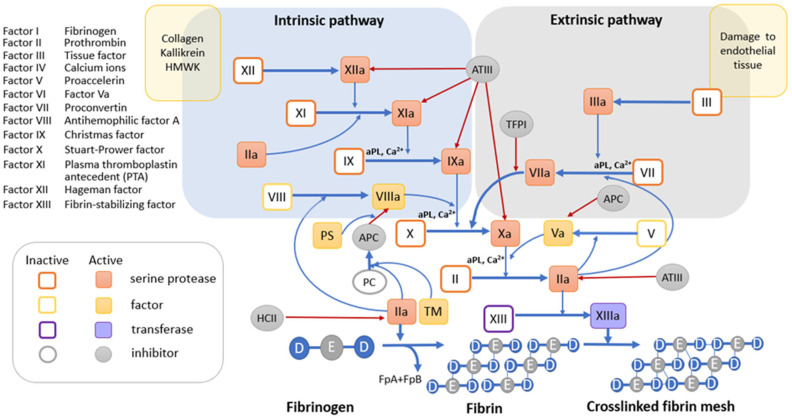
The coagulation cascade and its regulators. Coagulation is initiated via the extrinsic or intrinsic pathway. The extrinsic pathway initiates by exposure of tissue factor (FIII) and assembly of the extrinsic tenase, leading to prothrombinase and ultimate thrombin (IIa) production. Thrombin (IIa) is responsible for direct fibrin clot formation, further stabilized by FXIIIa. The intrinsic pathway is initiated by FXII interacting with negatively charged surfaces, autoactivation, and via kallikrein. Activated FXIIa activates FXI (FXIa), which activates FIX (FIXa) that binds FVIIIa, forming the tenase complex, where the intrinsic pathway converges with the extrinsic pathway. There are many interactions between components within this complex system. For example, thrombin can activate FXIII, FV, and FVII. Activation of the coagulation system is finely balanced and controlled through specific regulatory mechanisms, including activity of proteins such as antithrombin (ATIII), activated protein C (APC), heparin cofactor II (HCII), and tissue factor pathway inhibitor (TFPI).

The blood coagulation system is a series of sequential proteolytic reactions in response to damage to a vessel or some other stimulus, which results in the conversion of inactive proteins—blood clotting factors—into their corresponding proteases, which ultimately leads to the formation of a fibrin clot. It is important to note that all protease complexes of the blood coagulation system depend on Ca^2+^ ions and anionic phospholipids (aPL). One of the key sources of aPL are platelets. In response to vascular damage, platelet activation occurs. They bind to proteins at the site of damage and are activated and then aggregate. In this case, a regulated transfer of aPL occurs from the inner layer of the bilayer cell membrane to the outer one [28,29] and the release of the contents of the granules [30], which promotes coagulation. Platelets release important hemostatic components into the circulation including platelet activating factor, platelet factor 4 (PF4), P-selectin, adenosine diphosphate, and polyphosphate. They produce local cell-stimulating effects, recruit and activate neutrophils and monocytes, and may promote further availability of aPL, an important cofactor for the assembly of all coagulation cofactor/enzyme protein complexes. There are two ways to activate the formation of thrombin, a key factor in the blood coagulation system that catalyzes the formation of a fibrin clot. These are the extrinsic tissue factor TF pathway and the intrinsic contact pathway (Figure 2). 

#### 3.1.1. Tissue Factor Pathway 

Tissue factor (TF) is a transmembrane glycoprotein that is constitutively expressed by subendothelial cells and serves as a high-affinity receptor cofactor for FVII. When the vessel is damaged, TF becomes available for binding to FVII. This connection ensures autoproteolytic activation of FVII with the formation of the TF/FVIIa complex, called external tenase [31]. As part of this complex, TF accelerates the FVIIa-mediated conversion of the FX factor into its active form FXa by approximately 100,000 times [32]. Extrinsic tenase TF/FVIIa can also initiate activation of FIX [33] and FVIII [34]. FIXa and FVIIIa are a protease and its cofactor, respectively, that form the FIXa/FVIIIa complex called intrinsic tenase. Like extrinsic tenase, intrinsic tenase catalyzes the activation of FX. Once the concentration of FXa exceeds the threshold required to overcome the effects of circulating endogenous anticoagulants such as tissue factor pathway inhibitor (TFPI) [35] and antithrombin (AT), FXa activates its cofactor FV [36,37]. Assembly of the Ca^2+^-dependent prothrombinase complex FVa/FXa on a membrane containing aPL results in the cleavage of prothrombin to produce the potent serine protease thrombin [38]. Thrombin, in turn, triggers the polymerization of soluble fibrinogen by proteolytic conversion into cross-linked fibrin by thrombin-activated FXIII to produce a stable clot.

#### 3.1.2. The Internal Contact Activation Pathway 

The internal contact activation pathway is a critical link within the thromboinflammatory network, which is closely related to complement and coagulation. The intrinsic pathway is mediated by circulating factor FXII. Factor FXII constitutively exhibits low levels of activity [39]. Upon contact with a negatively charged surface, it catalyzes its own activation and that of plasma prekallekrein to form the plasma serine protease plasma kallikrein (Pka). To do this, it recruits the high molecular weight kininogen HK as a cofactor, which significantly accelerates the process [40]. Negatively charged surfaces for FXII may include damaged blood vessels, pathogens, DNA, RNA [41], neutrophil extracellular traps (NETs) [42], anionic polysaccharides, polyphosphates, activated endothelial cells, and platelets [43,44]. When there is enough FXIIa, FXI is activated. FXIa activates the formation of the intrinsic tenase FIXa/FVIIIa and ultimately the formation of fibrin by thrombin.

Thrombin recognizes several protein substrates at once, which contribute to its own generation and, as a consequence, amplification of the coagulation reaction [45]. It activates FV and FVIII and converts FXI to FXIa, the latter promoting the generation of its own tenase activity by further activating FIX. Combined with high levels of active Fva, increased FVIIIa/FIXa tenase activity significantly increases subsequent prothrombinase Xa/Va assembly and thrombin generation. At the same time, thrombin, in combination with its cofactor thrombomodulin, activates protein C. Activated protein C, associated with protein S, cleaves factors Fva and FVIIIa, thereby preventing excessive thrombin formation. Plasma kallikrein Pka, as part of the HK/Pka/FXIIa complex, cleaves HK to produce the proinflammatory bradykinin (BK) [46]. One of the functions of bradykinin is to activate the release of tissue plasminogen activator tPA from endothelial cells [47]. The formation of a complex of tPA with plasminogen (Pg) directly on the fibrin clot causes proteolysis of plasminogen to form the corresponding serine protease, plasmin. Plasmin breaks down fibrin, restoring blood flow.

### 3.2. Synergism in the Functioning of the Complement System and the Blood Coagulation System as a Key Factor in Thrombus Formation in HUS

**Figure 3:**
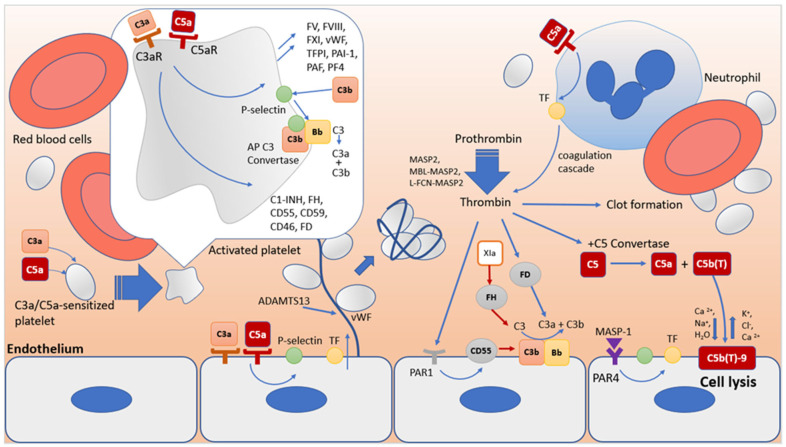
Complement and coagulation crosstalk. The complement and coagulation systems have common evolutionary origins. They exhibit several interactions that can affect activation, amplification and regulatory functions in both systems. Anaphylatoxins C3a and C5a, through their receptors, activate platelets sensitized to C3a and C5a, changing their adhesive properties and stimulating aggregation. Activated platelets secret FV, FVIII and FXI, fibrinogen, vWF, P-selectin, Pg, TFPI, PAI-1, PAF, PF4, as well as regulatory complement factors C1-INH, FH, CD55, CD59, CD46, FD, etc. Exposed P-selectin binds to its ligand C3b to ensure the assembly of alternative pathway C3 convertase. C5a triggers surface expression of TF by endothelial cells, monocytes, and neutrophils. Activated by C3a and C5a, endothelial cells express vWF, which can cause platelets aggregates formation. MASP2 protease, either alone or as part of the activated MBL-MASP2 and L-FCN-MASP2 complexes, is capable of stimulating fibrinogen metabolism and fibrin clot formation by cleaving prothrombin to form thrombin. Combined activity of thrombin and C5 convertase yielded C5a and C5b(T). C5b(T) forms the C5b(T)-9 complex with significantly higher lytic activity compared to C5b-9. Thrombin may also be able to enhance the C3 convertase assembly via activation of FD or, on the other hand, induce PAR1-mediated expression of complement decay accelerating factor (DAF), a membrane complement inhibitor.

As already mentioned, the complement system and the blood coagulation system have common activators and inhibitors that coordinate their relationships and activities (Figure 3) [27]. Thus, anaphylotoxins C3a and C5a, through their receptors, activate platelets sensitized to C3a and C5a, changing their adhesive properties and stimulating aggregation. As a result of platelet activation, factors contained in α-granules are released that modulate inflammation and coagulation, including blood coagulation factors FV, FVIII and FXI, fibrinogen, vWF, P-selectin, plasminogen Pg, TFPI, PAI-1, PAF, PF4, regulatory complement factors C1-INH, FH, CD55, CD59, CD46, FD, etc. [27]. During platelet activation, P-selectin, chondroitin sulfate A, and gC1q-R receptors are exposed on their surface. C1q, through the gC1q-R receptor, on the one hand, can trigger the classical pathway of the complement system [48]; on the other hand, it causes conformational changes in the GpIIbIIIa integrin, which supports platelet adhesion and aggregation [49]. Exposed on the surface of activated platelets, P-selectin binds to its ligand C3b to ensure the assembly of alternative pathway C3 convertase, which can be enhanced by properdin [50,51].

Endothelial cells also express C3a and C5a receptors [52,53,54], the interaction of which causes activation of leukocyte adhesion molecules, P-selectin, VWF, and TF [55], suppression of thrombomodulin [56], and damage to the glycocalyx [57]. In addition to endothelial cells, C5a also triggers the expression of TF on the surface of monocytes and neutrophils and the expression of the plasminogen activator inhibitor PAI-1 in mast cells. Normally, these cells express tissue plasminogen activator t-PA. An increased level of PAI-1 expression compared to t-PA leads to a change in the regulatory activity of mast cells from profibrinolytic to prothrombotic [58].

MASP2 protease, either alone or as part of the activated MBL-MASP2 and L-FCN-MASP2 complexes, is capable of stimulating fibrinogen metabolism and fibrin clot formation by cleaving prothrombin to form thrombin [59]. The MASP1 protease, although significantly lower in comparison with thrombin, has very similar activity. It cleaves the factor XIII A chain and the fibrinogen beta chain at sites identical to thrombin, but differs from thrombin in cleaving the fibrinogen alpha chain [60]. In addition, activated MASP1 can stimulate endothelial cells through the PAR4 receptor, which leads to the exposure of TF and P-selectin.

Finally, platelets, endothelial cells, and leukocytes are particularly sensitive to sublytic concentrations of C5b-9 (sC5b-9). The sC5b-9 complex induces the transbilayer flip of aPL, which is required for the activation of coagulation through the assembly of the corresponding tenases for the terminal generation of thrombin [61,62]. In turn, blood coagulation factors are also capable of activating the complement system at various stages. Thrombin, especially at high concentrations, cleaves C5 to form a fragment corresponding to anaphylatoxin C5a [63,64,65]. The combined activity of thrombin and C5 convertase leads to the formation of cleavage products C5a and C5b(T). In this case, C5b(T) forms the C5b(T)-9 complex with significantly higher lytic activity compared to C5b-9 [65], thereby enhancing the thromboinflammatory response to damage.

Thrombin can also enhance complement by acting through its own protease-activated receptors (PARs) on the plasma membrane. Thus, exposure of platelets to thrombin induces deposition of C3 and MAC [66,67]. Thrombin may also be able to indirectly enhance the assembly of C3 convertase through activation of FD [68,69]. On the other hand, thrombin induces PAR1-mediated expression of the complement accelerating factor DAF, a membrane inhibitor of the complement system [70]. Plasmin can also act as a regulator of the complement system.

Plasmin cleaves C3 and C5 to form anaphylatoxins C3a and C5a, but this does not lead to the formation of convertases, which may be due to the proteolytic activity of plasmin towards C3b and C5b [63,71,72]. Factors IXa, Xa, XIa, and PKa have been reported to cleave C5 bypassing true convertases in a C3-independent manner [63,64,73,74]. In turn, PKa can cleave FH and FB [75,76]. Thus, both systems are capable of amplifying each other’s activity and, after activation, require control by appropriate inhibitory mechanisms.

One of the key regulators of coagulation is antithrombin, the primary inhibitor of thrombin, FXa, and FIXa [77,78]. In the complement system, it inhibits MASP1 and MASP2 of the lectin pathway [79]. In turn, the complement system inhibitor C1-INH, which blocks several proteases, including C1r and C1s [80], MASP1 and MASP2 [81], and is also able to directly bind C3b, blocking the formation of C3 convertases, control the activation of PK in the hemostatic system and neutralizes PKa and FXIIa activity [82], and inhibit plasmin [83].

Tissue factor pathway inhibitor (TFPI), while an endogenous extrinsic tenase inhibitor [35,84,85], also inhibits the lectin pathway by preventing the MASP2 protease from cleaving factors C4 and C2 [86].

There is an evidence suggesting a potential coregulatory relationship between FH and FXIa [87]. FXIa degrades FH [88], reducing FH binding to endothelial cells, its cofactor activity in FI-mediated C3b inactivation, and its C3b/Bb degradation function. In turn, FH inhibits the activation of FXI by thrombin or FXIIa. A complex of FH with FXIIa was detected in plasma [89]. Evidence suggests that FH may promote ADAMTS13-mediated proteolysis of ULVWF to form monomers and dimers [90,91,92,93]. In turn, smaller forms of VWF are not only less amenable to C3b binding, but they may also act as a cofactor for C3b inactivation by FI [94].

Another regulator of the coagulation system, thrombomodulin, stimulates the production of CPB2 [95], which inactivates the proinflammatory mediators bradykinin, osteopontin, and the critical anaphylatoxins C3a and C5a [96]. Thrombomodulin also enhances FI-mediated inactivation of C3b in the presence of FH or C4b-binding protein [97,98,99].

Summarizing the above data, we can say that the biochemical pathways of the complement system and the blood coagulation system intersect and influence each other, have common cellular targets, and common pathways of activation and regulation. The activity of both systems is coordinated through complex feedback mechanisms, and the disruption of these mechanisms can lead to various severe complications, including the development of complement-associated thrombotic microangiopathies. Thus, in 5% of patients with aHUS, various heterozygous missense mutations in the thrombomodulin gene THBD were identified [97]. These mutations were found to be associated with a reduced ability to inactivate C3b and activate CPB2. Plasminogen deficiency variants are also associated with aHUS [100]. There are proposals to expand the panel of analyzed genes in patients with aHUS to include genes encoding factors of the blood coagulation system [100].

## 4. Bacterial Infections That Cause HUS

### 4.1. Hemorrhagic Shiga Toxin-Producing E. coli

Among the infectious agents that provoke the development of hemolytic uremic syndrome, hemorrhagic Shiga toxin-producing *E. coli* occupies a special place. STEC infections cause more than 2.8 million acute illnesses annually, with an average of 3890 cases of HUS, 270 cases of irreversible end-stage renal disease, and 230 deaths annually worldwide [101]. STEC-HUS primarily affects children, with peak incidence between 3 and 5 years of age [101]. The incidence rate in this age group reaches 10–17 cases per 100,000 children [102]. Symptoms appear 2–12 days after infection [102,103]. There is severe abdominal pain, bloodless diarrhea, accompanied by vomiting and fever. In 70% of cases, the disease progresses within 1–2 days and hemorrhagic colitis develops. Diagnosis of HUS occurs 6–10 days after the onset of diarrhea, when the first signs of acute renal failure are observed. Early dialysis has reduced mortality among STEC-HUS patients from 30% to 5% [104]. However, 25–30% of surviving patients subsequently experience renal and neurological complications [105,106].

#### 4.1.1. Pathogenetic Mechanisms of STEC-HUS

The pathogenetic mechanisms of the development of STEC-HUS are complex and affect a whole range of body systems. Once ingested, STEC colonizes the intestinal mucosa, tightly attaching to the enterocyte. This involves the destruction of microvilli, rearrangement of the enterocyte cytoskeleton, and accumulation of actin around the bacteria, forming a pedestal-like structure above the surface of the plasma membrane [107]. This contributes to diarrhea and intestinal inflammation. At this stage, to successfully colonize target organs, STEC can produce various virulence factors in the form of SPATE proteases, which inactivate both the complement system and the blood coagulation system. Thus, the serine protease Pic suppresses complement activation by proteolysis of C2, C3/C3b, and C4/C4b, interacts with FH/FI to inactivate C3b, and dearginates C3a, reducing its activity [108,109]. The EspP protease suppresses complement activation by proteolysis of C3/C3b and C5 and inactivates FV and α2-antiplasmin [110,111]. Also, the EspP protease can have a cytotoxic effect [112]. The metalloproteinase StcE cleaves the C1 esterase inhibitor C1-INH, thereby increasing its ability to neutralize C1 and MASP [113,114]. In addition to SPATE proteases, *E. coli* displays polyphosphate on its membrane to protect against complement. Polyphosphate prevents the assembly of MAC on the surface of the bacterial cell [115,116] and binds C1-INH, blocking the assembly of complement convertases. Thus, hemorrhagic *E. coli* has an arsenal of tools to defend against the complement system and maintain invasion. On the other hand, as mentioned earlier, polyphosphate can activate FXII by triggering the contact pathway to activate coagulation. It is generally accepted that the coagulation pathway triggered by FXIIa is not associated with ensuring hemostasis, because its deficiency does not lead to the development of bleeding [117]. Its activity is primarily associated with intravascular thrombosis [118,119].

Shiga-like toxin (Stx) released by STEC passes through the gastrointestinal epithelium and damages the blood vessels of the colon, causing bloody diarrhea [120]. Once in the bloodstream, it migrates along the bloodstream to organs whose cells express glycosphingolipid globotriaosylceramide [Gb3Cer] and globotetraosylceramide [Gb4Cer] receptors on their surface. These organs primarily include the kidneys, brain, and lungs [121]. Stx is known to have two isoforms, Stx1 and Stx2, which in turn are divided into subtypes [122]. The distinct mechanisms of action of Stx1 and Stx2 remain to be elucidated, but Stx2 is known to cause more severe symptoms [123].

The cytotoxicity of Stx can be enhanced by LPS in the outer membrane of Gram-negative bacteria by activating Stx receptors and increasing its inhibitory activity on protein synthesis in the cell [124,125]. Thus, in a mouse model, it was demonstrated that the combination of Stx and LPS induces a HUS-like response and changes in glomerular endothelial cells that are different from those observed with exposure to Stx or LPS alone [126,127,128]. Thus, Gram-negative bacteria may increase Stx-induced inflammation and damage to internal organs.

All Stx consist of one biologically active A subunit (32 kDa), which is associated with five B subunits (7.7 kDa) [129]. This structure allows the bacterium to safely secrete the toxin and ensures binding to cells expressing the Gb3Cer/CD77 or Gb4Cer receptors [130]. After interaction with the receptor, the toxin undergoes endocytosis and retrograde transport to the Golgi apparatus and endoplasmic reticulum [131]. In the endoplasmic reticulum, subunit A is proteolytically cleaved to form the A1 fragment (27 kDa). This fragment moves into the cytosol and interrupts protein synthesis, inactivating ribosomes by cleaving off an adenine residue from 28S ribosomal RNA [132]. Caused by impaired protein synthesis, ribosomal stress triggers a variety of signaling pathways that can initiate a pro-inflammatory response (cytokines and chemokines) and apoptosis [133,134,135,136].

Both Stx1 and Stx2 are found in the serum of HUS patients [137,138]. Stx circulates in the blood plasma as a complex with cells, or in microvesicles released by blood cells. Possible carriers of Stx are erythrocytes, platelets, and monocytes, which have the Gb3 receptor on their surface [139], as well as neutrophils, which do not express the Gb3 receptor, but interact with Stx through Toll-like receptor 4 (TLR4) [140]. It has been suggested that microvesicles released by blood cells are a means of transporting Stx to glomerular cells [103,141]. Stx can be incorporated into blood microvesicles generated by neutrophils, monocytes, platelets, and red blood cells, translocate to target cells, and be taken up by endocytosis [141,142]. Thus, Stx, interacting at the first stage with its receptors and penetrating into blood cells, can subsequently leave them as part of microvesicles and penetrate into cells that do not carry specific receptors on their surface [141,142]. In addition to the Stx toxin, the contents of microvesicles released by platelets and monocytes may contain various factors, such as activated complement components or tissue factors, and also be transferred to target cells [142].

Damage to the glomerular endothelium has been reported as one of the primary events in the development of thrombotic microangiopathic lesions in STEC-HUS [136].

Under the influence of Stx in endothelial cells, nuclear transcription factor-κB (NF-κB), proinflammatory cytokines and chemokines, cell adhesion molecules are activated [128,143,144,145]. In this case, endothelial cells lose their thromboresistant phenotype and demonstrate increased adhesive abilities. Stx has been demonstrated to induce the formation of platelet thrombi on the surface of endothelial cells under conditions of high shear stress, similar to what is observed in the microvascular bed [146]. In this case, von Willebrand factor acts as a factor directly involved in the formation of a platelet thrombus. It has been demonstrated that Stx can directly bind to von Willebrand factor on the cell surface, protecting it from degradation by the metalloproteinase ADAMTS13 [147] Blockade of adhesion proteins, including P-selectin, reduced the thrombogenic effect of Stx [146].

Podocytes can also be subject to cytotoxic effects of Stx [148,149,150,151]. Upon binding to Gb3 receptors, Stx activates p38 and p42/44 mitogen-activated protein kinases (MAPK), as well as the transcription factors NF-kB and AP-1 in human podocytes, causing the release of cytokines such as IL-1 and TNF-α. In turn, cytokines, through increased expression of Gb3, increased the sensitivity of cells to the toxin [150,151,152] and favored apoptosis [148].

In addition to the direct cytotoxic and proinflammatory activity of Stx and other waste products of *E. coli*, the complement system plays an important role in the pathogenesis of STEC-HUS [153,154,155,156].

#### 4.1.2. The Role of the Complement System in the Pathogenesis of STEC-HUS

**Figure 4:**
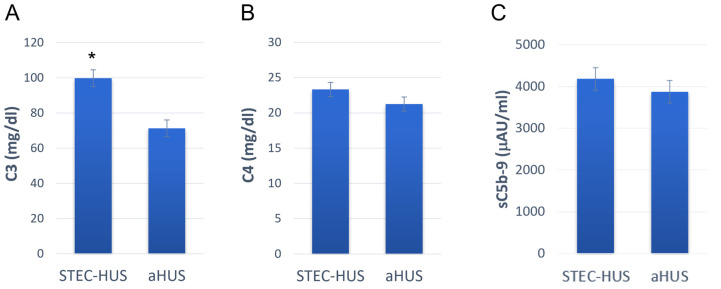
Complement system activation in STEC-HUS and aHUS. (**A**)—C3 level in plasma of aHUS patients was lower than C3 level in plasma of STEC-HUS patients. (**B**)—C4 levels in plasma of STEC-HUS patients and aHUS patients were within normal limits and did not differ significantly from each other. (**C**)—sC5b-9 level was increased in plasma of STEC-HUS and aHUS compared to the norm. Results represent the mean  ±  standard error of the mean. (* *p* < 0.01 by independent-samples *t*-test).

Already, the first attempts to investigate the participation of the complement system in the pathogenesis of STEC-HUS revealed decreased levels of C3 and increased levels of its breakdown products C3a, C3b, C3c, and C3d in the blood serum of children during the active phase of the disease [157,158,159,160]. A link between levels of C3 in blood plasma, leukocytosis, and the severity of the disease was established. At the same time, fluctuations in levels of C3 were noted in the range from 68 mg/dL to 108 mg/dL, with a norm of 88–169 mg/dL [160]. These data are consistent with the preliminary results we obtained from our preliminary studies. The average amount of C3 in the blood plasma of the STEC-HUS patients we examined was 99.8 ± 4.7 mg/dL (mean ± SEM, *n* = 9) (Figure 4). In some cases, C4 levels were decreased in patients with STEC-HUS [160], but in most cases, they remained within the normal range, limited to the range of 12–33 mg/dL [159]. In the group of STEC-HUS patients we examined, the amount of C4 in the blood plasma was also within normal range and averaged 23.3 ± 2.8 mg/dL (mean ± SEM, *n* = 9) (Figure 4). At the same time, in patients with STEC-HUS, increased Bb levels were observed, which directly indicates the activation of the alternative pathway of the complement system [161]. Subsequent studies showed that an increase in the level of Bb in the plasma of patients is accompanied by an increase in the level of soluble C5b-9, correlating with it and the severity of oliguria [162]. We also noted increased levels of soluble C5b-9 in patients with STEC-HUS. The average level of soluble C5b-9 in the group of STEC-HUS patients we examined was 4185 ± 274 μAU/mL, with the established norm being considered <1000 μAU/mL (Figure 4). We also compared the plasma levels of C3, C4, and soluble C5b-9 in STEC-HUS patients with those in aHUS patients and found that with similar levels of C4 and soluble C5b-9, aHUS patients demonstrate a more pronounced decrease in C3 levels (Figure 4). On the one hand, it can be assumed that the activation of the alternative pathway in patients with STEC-HUS is less pronounced. On the other hand, other pathways of complement activation may be involved in the pathogenesis of STEC-HUS. An increase in C3a levels was also found in the acute phase of the disease and a return to normal levels after recovery [163]. This fact supports the hypothesis that complement plays an important role in the pathogenesis of STEC-HUS.

In addition to the activation of the complement system, deposition of C3 and C5b-9 along with fibrin accumulation is noted in the glomeruli of children with STEC-HUS [163]. Taken together, these data suggested a connection between activation of the complement system, renal microvascular thrombosis, and damage to glomerular podocytes.

#### 4.1.3. Pathways of Complement Activation in STEC-HUS

The data presented in the literature allow us to identify several possible pathways of activation of the complement system in STEC-HUS. One of them may be the direct interaction of Stx with complement factors. It has been demonstrated that Stx2, when added to normal human serum, activates the complement system in the fluid phase, causing the formation of soluble C5b-9 [164]. It is assumed that Stx is able to directly interact with complement proteins and activate the alternative pathway. The ability of Stx2 to bind to short domains (SCRs) 6–8 and 18–20 of factor H, which are responsible for recognition of the host cell surface, has been revealed [164]. Stx2 binding to factor H disrupted its cofactor activity on the cell surface, leading to increased complement activation and accumulation of C3b on the cell surface, but did not affect factor H activity in the fluid phase [164]. In addition to binding factor H, Stx2 also acts as a ligand for two other factor H family proteins, FHR-1 and FHL-1, which share amino acid sequence and regulatory function with factor H [165]. In addition to direct activation of the complement system, Stx can indirectly change its activity. Thus, Stx2 has been shown to modulate the expression of CD59, a membrane-bound complement regulator that inhibits the formation of the C5b-9 complex. Endothelial cells exposed to Stx2 showed a decrease in CD59 mRNA levels and its surface expression [166]. Abnormal activation of the alternative pathway can also be achieved through activation of factors B and D by MBL/ficolin-associated serine proteases (MASPs), suggesting the possibility of indirect activation of the alternative complement pathway [167]. Recent evidence suggests that the lectin pathway plays a role in the disease, as inhibition of MBL2 in Stx-HUS mice significantly limits renal C3d deposition and damage [168]. The mechanisms of lectin pathway activation are unclear. Finally, activation of the complement system may be mediated by P-selectin. In this case, the complement system closely interacts with the blood coagulation system according to the principle of mutual reinforcement. A number of observations support this assumption. Stx, by binding to its specific endothelial receptor Gb3, modifies endothelial thromboresistance. Exposure of human umbilical vein endothelial cells HUVEC and glomerular microvessels GMVEC to Stx stimulated exocytosis of Weibel–Palade bodies containing both proinflammatory factors (P-selectin, eotaxin-3, IL-8, angiopoietin-2, CD63, α1,3-fucosyltransferase VI, osteoprotegerin) and hemostasis factors (von Willebrand factor, t-PA) [169]. P-selectin on the surface of endothelial cells is able to bind C3 and activate the alternative complement pathway. In turn, whole blood perfusion of endothelial cells that were treated with Stx1, compared to controls showed increased C3 deposits and a larger area of thrombus formation [170]. Moreover, the addition of the soluble complement receptor sCR1, a C3 inhibitor, as well as the addition of antibodies blocking P-selectin, completely suppressed the formation of blood clots on the surface of endothelial cells, which indicates a functional connection between C3 deposits on the endothelial surface and thrombosis [170]. Apparently accumulated as a result of P-selectin-mediated complement activation, C3a through the C3aR receptor enhances the expression of P-selectin and t-PA-dependent cleavage of thrombomodulin TM, and activates platelets, thereby stimulating both the complement system and the blood coagulation system [170,171]. Ultra-high molecular weight von Willebrand factor (ULVWF) multimers on the surface of endothelial cells bind and activate platelets [172], triggering a series of events leading to activation of the blood coagulation system and stimulation of the complement system. It was found that Stx1 and Stx2 inhibit the cleavage of ULVWF by the metalloproteinase ADAMTS13 [172]. It has also been shown that Stx-1, in combination with TNFa, disrupts the expression, synthesis, association, and secretion of TFPI, and increases the level of functional TF on the surface of endothelial cells [173]. Thus, it can be assumed that there is synergism in the functioning of the complement system and the blood coagulation system in the pathogenesis of STEC-HUS.

#### 4.1.4. Targets of the Complement System in the Pathogenesis of STEC-HUS

The range of target cells of the complement system in STEC-HUS is quite wide. In addition to the endothelium, deposits of C3 and C5b-9 have been observed on the surface of blood cells and the microvesicles they secrete in patients with STEC-HUS [160,174]. Exposure of whole blood to Stx2 causes the formation of platelet-monocyte and platelet-neutrophil aggregates with surface-bound C3 and C9 [160]. In turn, the deposition of complement activation products on platelets and monocytes leads to the release of TF-expressing microparticles that may contribute to the development of thrombosis in STEC-HUS [175,176]. Exposure of erythrocytes to Stx2 causes the release of hemoglobin and the formation of C3 and C5b-9 coated microvesicles [174]. Interestingly, Stx2-induced hemolysis was not apparent in the absence of plasma and was inhibited by heat inactivation, as well as by eculizumab, the purinergic P2 receptor antagonist suramin, and EDTA. Release of microvesicles by erythrocytes in whole blood in the presence of Stx2 was not observed in the absence of factor B and was suppressed by EDTA and P2 purinergic receptor antagonists [174]. These observations directly indicate the involvement of the alternative complement pathway in the hemolytic process occurring in STEC-HUS [174].

C3 and C5b-9 deposits are also found in the glomeruli of STEC-infected mice [163]. In wild-type mice with Stx2/LPS-induced HUS, C3 deposition was accompanied by fibrin(ogen) accumulation, dysfunction, and loss of podocytes [128,170,177]. Complement accumulation in the glomerulus has been shown to activate important regulators of podocyte adhesion, migration, and intercellular communication, such as integrin-linked kinase (ILK) signaling, as well as the Snail transcription factor, which is responsible for nephrin suppression [177]. However, in factor B-deficient mice, complete restoration of glomerular architecture was observed after administration of Stx2/LPS, which clearly indicates that complement activation through the alternative pathway contributes to podocyte dysfunction [177].

#### 4.1.5. Immunometabolic Alterations in STEC-HUS

Immunometabolism is a complex interplay between immune and metabolic path-ways. Its dysregulation is considered to be one of the reasons for acute (AKI) and chronic (CKD) kidney disease [178]. Immunometabolic system includes dendritic cells, macrophages, T and B cells, the messengers of immune response (cytokines and chemokines, etc.), their membrane receptors, and intracellular metabolic processes that are regulated by these receptors [179]. It is an integral part of systemic metabolism. In a healthy body under normal physiological conditions, immunometabolic processes are tightly regulated. However, as shown in review [178], their imbalance occurring under the action of pathogenic factors is associated with the development of kidney diseases such as lupus nephritis, diabetic kidney disease, and polycystic kidney disease. Below, we will consider immunometabolic alterations in the most common kidney disease in children typical haemolytical uremic syndrome and their possible connection with complement activation.

It is known that pro-inflammatory cytokines produced by a multiprotein complex NLRP3 inflammasome are drivers of diabetic kidney disease [180]. NLRP3 inflammasome is involved also in lupus nephritis polycystic kidney disease. Persistent or dysregulated activation of the NLRP3 inflammasome has been implicated in the development of glomerulonephritis, diabetic nephropathy, tubulointerstitial nephritis, and other inflammatory renal disorders.

In typical HUS, the level cytokines is elevated [181]. An increase in cytokines can be caused directly by Shiga toxin Stx2 [182]. Experiments in vitro with peritoneal macrophages (PMs) isolated from wild-type (WT) C57BL/6J mice and gene knockout mice (Nlrc4–/–, Aim2–/–, and Nlrp3–/–), and in vivo with WT mice and Nlrp3–/– mice indicate that Stx2 activates their production of IL-1β [183]. This work shows that Oridonin and other Nlrp3 inhibitors significantly impair the survival of mice treated with Stx2.

Activation of Nlrp3 in children with STEC-HUS may occur as a result of complement activation. It is known that NLRP3 inflammasome activation is caused by peptides C5a [183] and C3a [184], and can be promote by sublytic deposition of MAK [185,186].

### 4.2. Shigella dysenteriae

In addition to hemorrhagic strains of *E. coli*, the development of HUS can be caused by infection with *S. dysenteriae*. Compared to *E. coli*, this infection is significantly less widespread on Earth and is represented mainly in developing countries in Africa and Asia. This is largely due to the mechanisms of infection transmission. The only carrier of *S. dysenteriae* is humans, and transmission of infection occurs through the fecal–oral route through contaminated food and objects. In this regard, the frequency of HUS associated with *S. dysenteriae* is significantly lower than HUS associated with *E. coli* [187]. Thus, between 1987 and 2012, only 488 cases of shigellosis-associated HUS were documented. However, the average mortality rate is significantly higher than with STEC-HUS and can reach 59% [188,189,190,191,192,193,194]. It is HUS that is the main cause of death in dysentery epidemics caused by *S. dysenteriae* infection [187]. The development of HUS in shigellosis is predominantly associated with infection with *S. dysenteriae* serotype SD1 [195]. This serotype, in comparison with other Shigella species and *S. dysenteriae* serotypes, is characterized by an increased level of Shiga toxin secretion. This toxin is identical to the Stx1 toxin of the hemorrhagic *Escherichia coli* O157:H7, which, as previously mentioned, together with Stx2 plays a key role in the development of HUS [196]. In addition to Shiga toxin, *S. dysenteriae* serotype SD1, like *E. coli*, produces LPS, which is considered a potential mediator of vascular damage, the development of DIC, and sepsis. LPS was detected in the blood of patients with HUS associated with infection with *S. dysenteriae* serotype SD1. At the same time, in patients with diarrhea uncomplicated by the development of HUS, LPS was rarely detected in the blood [197]. Double intravenous administration of LPS produced by *S. dysenteriae* caused a generalized Schwartzman reaction and the clinical picture of HUS [198]. These facts suggest that HUS caused by *S. dysenteriae* infection and HUS caused by *E. coli* O157:H7 infection share common developmental mechanisms. However, it is worth considering that infection with *E. coli* O157:H7 can cause the development of HUS in children and adults, while shigellosis provokes the development of HUS exclusively in children. Also, unlike *E. coli*, *S. dysenteriae* serotype SD1 is enteroinvasive. The diarrhea it causes is accompanied by the penetration of bacteria into the general bloodstream.

### 4.3. Streptococcus pneumoniae and Other Neuraminidase-Producing Bacteria

The second most common infectious agent after hemorrhagic *E. coli* that can cause hemolytic uremic syndrome is *S. pneumoniae*, which, according to some data, accounts for approximately 5% of all cases of HUS in children. Other sources estimate that pneumococcal HUS (SP-HUS) accounts for 38% to 43% of all non-STEC HUS cases. The annual incidence is approximately 6 cases per 10 million children under 18 years of age per year. Most often caused by infection with *S. pneumoniae*, HUS develops after pneumonia complicated by empyema or meningitis.

To date, several probable mechanisms for the development of HUS in pneumococcal infection are being considered. The most widely accepted opinion is that neuraminidase produced by *S. pneumoniae*, circulating in the bloodstream, removes N-acetylneuraminic acid (Neu5Ac) from the sialoglycoproteins of cell membranes from the surface of red blood cells, platelets, and endothelial cells, exposing the Thomsen–Friedenreich antigen (T-antigen) [199]. This disaccharide is not detected on the surface of normal cells and, if exposed on the cytoplasmic membrane, is recognized by immunoglobulins circulating in the blood [200]. Antibodies bind T-antigen on the surface of erythrocytes, platelets, and endothelial cells, which leads to agglutination of erythrocytes, platelet aggregation in the microvasculature and the development of TMA [201,202]. In addition to the presentation of T-antigen on the cell surface, desialylation can lead to the loss of factor H binding sites by cells. This may result in uncontrolled activation of the alternative complement pathway. It has been shown that in patients with SP-HUS, factor H itself is also desialylated, which, on the one hand, increases its C3b binding activity, and, on the other hand, does not in any way affect its ability to dissociate C3b and C3bBb(P) convertase and reduces its ability to inhibit red blood cell agglutination [203]. However, desialylation of erythrocytes and platelets is not specific for HUS. This process is also observed in patients with invasive pneumococcal infection not burdened by HUS [202,204]. Potential targets for desialylation in the pathogenesis of SP-HUS are not limited to complement factors and cell membrane sialoglycoproteins. Desialation can also affect factors of the blood coagulation system, changing their functional activity. Thus, the most important point in the activation of platelet aggregation by neuraminidase is the desialylation of Factor VIII [205]. This mechanism excludes the role of mutations in the genes of complement factors as a determining risk factor for the development of SP-HUS, although it is noted that desialylation may lead to a decrease in cell resistance to the activity of complement factors [199,206,207]. Fibrinogen desialylation reduces thrombin time in patients with liver disease, which is apparently due to increased aggregation of fibrin monomers [208,209]. Desialylation of plasma von Willebrand factor induces its binding to platelets [210]. Desialation of FVIIa promotes its active recognition by hepatocytes through the asialoglycoprotein receptor and accelerates its clearance from the bloodstream [211]. The coagulation activity of factor IX is markedly reduced when sialic acid residues are removed [212]. Plasma clearance of prothrombin is enhanced by desialylation. At the same time, the question about changes in its activity remains open [213,214,215]. It can be assumed that desialylation of factors of the blood coagulation system can affect both their procoagulant/anticoagulant activity, exacerbating the prothrombotic state, and on regulatory activity in relation to the complement system.

HUS caused by pneumococcal infection can develop in the absence of antibodies to T antigen [216]. Only 60–90% of patients with HUS show a positive Coombs test [217] Dysregulation of the alternative complement pathway may be caused by direct binding of factor H to bacterial proteins. Thus, some serotypes of *S. pneumoniae* express proteins (PspC, CbpA, Hic) that can bind factor H, changing its activity and eliminating it from the blood plasma [218,219]. Genetic studies have demonstrated the presence of mutations previously associated with aHUS in a significant proportion of patients with SP-HUS [203].

The list of bacteria capable of producing neuraminidase and causing HUS is not limited to *S. pneumoniae*. To date, cases of the development of HUS due to a bacterial infection caused by β-hemolytic streptococcus group A (*S. pyogenes*) have been described [220,221,222]. In this case, hypocomplementemia and C3 deposits on the surface of capillary endothelial cells were observed [221,223]. In addition to neuraminidase, the M1, Fba, and NAPlr proteins produced by *S. pyogenes* may be involved in the pathogenesis of HUS [224,225]. By binding factor H of the complement system, the Fba protein can disrupt its regulatory activity. Bacterial proteins of the M family are able to bind the protein S complex with C4BP [226], thereby affecting its balance in plasma and regulation of the blood coagulation system. It was also found that *S. pyogenes* is capable of activating the contact pathway of activation of the blood coagulation system, binding fibrinogen through the M1 protein and forming a fibrin network on the surface of the epithelium [227]. Fibrinogen breakdown products can contribute to serious complications by causing the release of monocyte/macrophage-derived interleukins 1 and 6 (IL-1, IL-6), and plasminogen activator inhibitor 1 (PAI-1). While IL-1 and IL-6 cause additional damage to the vascular endothelium, PAI-1 inhibits fibrinolysis and subsequently accelerates further thrombus formation. The nephritis-associated plasmin receptor NAPlr is deposited in glomeruli and causes glomerular damage by binding plasmin and maintaining its local activity [228,229]. In addition to streptococci, other bacteria that produce neuraminidase, such as *Clostridium perfringens*, can cause HUS [230,231,232,233].

### 4.4. Bordetella pertussis

*B. pertussis* is the causative agent of the acute respiratory infectious disease known as whooping cough. Today, whooping cough remains a significant cause of morbidity and mortality in children under 2 years of age. The infection is transmitted by airborne droplets and manifests itself by damage to the mucous membranes of the upper respiratory tract. In particularly severe cases, the infection can be accompanied by the development of complications such as seizures, apnea, pneumonia, or encephalopathy, and lead to death [234]. The first case of HUS developing due to *B. pertussis* infection was described in 2002 [235]. A child with abnormalities in the structure of factor H developed HUS several weeks after infection, followed by death. Subsequently, several cases of the development of non-recurrent HUS after infection with *B. pertussis* were described in patients without defects in complement factors [236,237,238]. It was found that in patients with non-recurrent HUS, C3 and C4 levels may decrease simultaneously in the acute phase [238]. This suggests simultaneous activation of the classical and alternative complement pathways. The activation mechanisms have not yet been fully established. *B. pertussis* is known to express a number of proteins that allow it to evade the activity of the complement system [239]. The BrkA autotransporter protein suppresses the deposition of C3 and C4 and the formation of MAC on the bacterial surface. The mechanism of this suppression is unknown. BrkA is proposed to either promote C4b degradation or inhibit C4 activation [240]. The Vag8 protein has been identified as a C1 inhibitor binding factor on the bacterial cell surface [241,242]. The structure of Vag8-C-INH shows a unique mechanism of C1-INH inhibition, where Vag8 sequesters the reactive center loop of C1-INH, preventing its interaction with the target proteases [243]. C1-INH is a key inhibitor of serine proteases of complement and coagulation systems. Its inactivation can lead to aberrations in these systems’ functions. The surface protein filamentous hemagglutinin (FHA), in complex with one or more as yet unidentified BvgAS-regulated proteins, binds the complement regulatory factor C4BP [244,245,246]. C4BP binds to C4b, thereby dissociating the CP/LP C3 convertase C4b2a, and acts as a cofactor for plasma protease factor I in the proteolytic degradation of C4b [247]. Finally, *B. pertussis* bacteria are able to bind complement factor H and similar proteins FHR-1 and FHL-1 on their surface. Factor H binding has been shown to occur through its SCR20 and SCR5–7 domains [248,249]. On the one hand, the binding of regulatory proteins allows the bacterium to evade the activity of the complement system. On the other hand, this process can upset the balance in the regulation of the complement and coagulation systems, leading to its excessive activities.

### 4.5. Salmonella typhi

*S. typhi* infection is extremely rare as a cause of the development of HUS, and therefore the mechanisms underlying the development of HUS in typhoid fever remain unclear. However, it is assumed that the lipopolysaccharide of *S. typhi* may act as a key factor triggering the chain of interactions leading to the development of HUS. Immunoglobulins IgM and IgA to *S. typhi* are detected in the plasma of patients with typhoid fever burdened with HUS [250] The lipopolysaccharide antigen Salmonella O-Ag was found to activate the alternative pathway of complement activation [251,252]. In turn, the Vi capsular antigen protects bacteria from phagocytosis and complement-mediated lysis by partially but not completely suppressing opsonization, probably by shielding the O antigen [253,254]. Thus, *S. typhi* bacteria can activate the alternative pathway of the complement system, maintaining their viability for a relatively long time. On the other hand, within permissive macrophages, Salmonella increases the expression of the outer membrane protease PgtE, which degrades various complement components, including C3, C3b, C4, C4b, C5, factor B, and factor H, mediating serum resistance [255,256,257]. PgtE activity is not limited by complement factors. PgtE can cleave vitronectin, suppressing MAC resistance [249]. Blood coagulation factors can also act as a substrate of PgtE. Thus, PgtE activates plasminogen and cleaves PAI-1, TAFI, and α2-antiplasmin [255,258,259] Thus, one could talk about the activation of fibrinolysis in infected *S. typhi*, but clinical data suggest the opposite. In those infected, activation of the blood coagulation system is observed. There is an increase in the level of thrombin and fibrinogen against the background of a decrease in the levels of protein C and antithrombin. Patients showed signs of suppressed fibrinolysis and marked activation of endothelial cells [260]. Perhaps the role of PgtE in the regulation of the complement system is not so significant, or the range of its functions is wider than currently assumed. One way or another, the mechanisms of HUS development in *S. typhi* infections remain unknown and require research.

### 4.6. Other Bacterial Infections

In the modern literature, there is increasing evidence that some bacteria that have not previously produced Stx can acquire this ability. Thus, different groups of researchers discovered *Shigella sonnei* strains producing either Stx-1 or Stx-2a [261,262,263,264] It is known that *stx* genes are carried in the genome of λ phages or “*stx*-converting bacteriophages”, which can insert DNA into the chromosomes of host bacteria through transposition or recombination [264]. *E. coli* and *Shigella* spp. are close genetic relatives, allowing *Shigella* species to obtain toxin genes from *E. coli*. This indicates that previously nontoxigenic *S. sonnei* can acquire virulence genes upon invasion by a phage carrying the toxin genes. Phages carrying the *stx* gene have been detected in wastewater and in the feces of healthy people [265,266]. These phages have been found to be able to infect and replicate in cultures of *Stx*-negative strains of *E. coli* and *S. sonnei* [265]. Phages carrying the *stx* gene remain viable longer than their bacterial hosts and can enter the body with contaminated food and water [267]. Another way to convert bacteria that do not normally produce Stx (such as *S. sonnei*) into Stx producers involves co-infection with hemorrhagic strains of *E. coli* or *S. dysenteriae*. As a result, bacteriophages released during lysis of *E. coli* or *S. dysenteriae* infect bacteria that do not carry *stx* genes [265,268]. To date, at least one case of HUS secondary to *S. sonnei* infection has been described [269].

## 5. Viral Infections That Cause HUS

### 5.1. Influenza Virus

Influenza remains one of the most common infectious causes of death in the Western world, with high genetic variability and emerging resistance to antiviral drugs [270]. Influenza A, B, and C viruses pose a threat to humans [271]. The literature describes cases of the development of hemolytic uremic syndrome against the background of an acute respiratory infection caused by the influenza A virus, in particular H1N1 [272,273,274,275,276]. Several cases of HUS caused by influenza B virus have also been reported [277,278,279]. There are relatively few such clinical cases and, at this stage, the mechanisms mediating the appearance of HUS with the influenza virus remain poorly understood. Both in vitro and in vivo experiments have demonstrated the ability of the H1N1 virus to cause endothelial cell apoptosis, platelet activation, and subsequent microthrombi formation [270,280,281]. The pathogenicity of influenza A virus as an inducer of HUS may be based on the presence of neuraminidase (NA), encoded in the sixth RNA segment and forming a tetramer on the outer membrane of the virus. NA has sialidase activity necessary for virus exit from the cell. The participation of influenza A virus neuraminidase in the pathogenesis of HUS may differ from that in *S. pneumonia* infection due to the fact that in the case of pneumococcal infection, neuraminidase is in a free state and circulates in the blood plasma, while the virus neuraminidase is directly associated with its lipoprotein envelope. It has been demonstrated that red blood cells that have been pre-exposed to viral particles activate the alternative complement pathway in human serum. Moreover, the level of APC activity depended on the number of viral particles to which erythrocytes were preliminarily exposed and on the degree of desialylation of erythrocyte membranes [282]. In patients with influenza A H1N1, activation of the complement system is observed, expressed in an increase in the level of C5b-9 in the blood plasma [283]. It has also been demonstrated that acute lung injury in mice infected with influenza A H5N1 is associated with excessive complement activation with deposition of C3 and C5b-9 and increased expression of complement receptors C3aR and C5aR [284]. It has been suggested that the pathogenesis of influenza virus-induced HUS may involve activation of adhesion molecules on the surface of endothelial cells, as has been demonstrated in the case of STEC-HUS, but this assumption needs to be tested. A hypothesis has also been expressed about the triggering effect of the virus on the HUS-sensitive haplotype [285,286,287]. Thus, in some patients with thrombotic microangiopathy associated with influenza A and B, mutations potentially associated with dysregulation of the complement system (C3 and MCP mutations) were found [273,277,278,288]. On the other hand, there have been cases where activation of the complement system and the development of TMA were observed in influenza-infected patients who did not carry mutations [289]. However, this fact does not allow us to exclude the hypothesis about the direct role of the H1N1 virus in the determination of transient activation of complement and the development of HUS.

In addition to the activation of the complement system, the activation of the blood coagulation system is observed during H1N1 infection. Thus, it was found that particles of the H1N1 virus in the presence of blood serum are capable of activating platelets in two ways at once. The first pathway involves the formation of the H1N1/IgG immune complex and subsequent recognition of this complex by the platelet FcγRIIA receptor. The second pathway is mediated by complement-independent activation of thrombin [280]. Ultimately, the activation of platelets leads to their release of microparticles with subsequent activation of both the blood coagulation system and the complement system.

### 5.2. Human Immunodeficiency Viruses (HIV)

The association between HIV infection and the occurrence of HUS was documented as early as 1984 [290]. However, the role of HIV in the described case was ambiguous. The patient was undergoing treatment for Kaposi’s sarcoma at the time of development of HUS and died from sepsis caused by *Staphylococcus aureus* infection. Subsequently, cases of the development of HUS against the background of HIV infection, not burdened by other infectious and tumor diseases, were described. On the one hand, a study of HUS patients with HIV infection showed that the development of the syndrome is not necessarily due to the presence of mutations associated with HUS. On the other hand, the data obtained indicate that the complement system is involved in the pathogenesis of HUS [291,292]. Thus, in a woman with established HIV infection, HUS was accompanied by a decrease in the level of factor C3 and factor B of the complement system. C4 levels remained within normal limits, indicating activation of the alternative complement pathway. Kidney biopsy showed colocalization of elevated serum MAC, C5b-9 deposits, and arteriolar microangiopathic lesions in the kidney. However, the use of the alternative pathway inhibitor of the complement system eculizumab was effective and led to remission. It is worth noting that the researchers were not able to test the patient for mutations associated with complement regulatory proteins [291]. Genetic examination of another patient with HIV-associated HUS did not reveal the presence of a single mutation associated with disturbances in the activity of the complement system. Factor H autoantibodies were tested negative. ADAMTS13 levels are within normal limits (72%). However, this patient was prescribed eculizumab therapy, which gave a positive result. It is logical to assume that, in this case, HUS was triggered by HIV through activation of the alternative complement pathway [292]. However, studies in a group of asymptomatic patients with HIV infection and patients with sepsis and malaria associated with HIV infection showed that HIV can activate the complement system through the classical pathway [293].

The inconsistency of the data obtained by different groups of researchers may be due to the presence of concomitant diseases and mutations associated with aHUS in patients with HIV infection, as well as at what stage of development of the immunodeficiency caused by HIV infection the patient was at the time of examination [294]. Nevertheless, several possible mechanisms can be identified, the launch of which can lead to activation of the complement system and disturbances in its functioning, leading to the development of TMA. Extensive evidence indicates that during HIV infection, antibody-mediated activation of the classical pathway occurs, leading to complement-mediated lysis of viral particles and virus-infected cells [295,296,297]. Lysis has been found to be rapid and efficient in the early stages of infection [294]. Antibody levels rise in response to gradually increasing viral load and appear to remain high throughout the disease [298]. As a consequence, increased activity of the classical complement pathway remains.

Activation of the complement system by the HIV-1 virus can also be mediated by the lectin pathway [299]. The ability of the gp120/gp41 Env complex to bind mannan-binding lectin (MBL) and activate the complement system has been demonstrated [300,301,302]. In addition, in vitro experiments have shown that MBL can influence the clearance of HIV-1 from the blood by binding the virus, followed by uptake by tissue macrophages, and enhancing antibody-mediated neutralization [303,304]. However, the role of the lectin pathway of the complement system in the development of the immune response to HIV infection requires further research.

Activation of the complement system during HIV infection is accompanied by opsonization of viral particles by complement system factors, which, on the one hand, protect them from complement-mediated lysis and, on the other, enhance their virulence. Deposition of C3 cleavage products and anaphylatoxin C5a on HIV-1 viral particles facilitates the interaction of HIV-1 with cells such as monocytes/macrophages and dendritic cells expressing complement receptors CR3 and CR4 [305,306]. Opsonization of intact viral particles by C1q results in enhanced HIV-1 infection in cell cultures, which is mediated by CR2 receptors [307]. CR1 and CR2 have been shown to contribute in an independent and complementary manner to the entry of opsonized virus into T cells expressing the complement receptor [308]. It has also been demonstrated that complement activation products C5a and C5a(desArg), but not C3a or C3a(desArg), can increase the susceptibility of monocytes and macrophages to HIV infection by stimulating the secretion of TNF-alpha and IL-6 by these cells [309]. In addition to activating the complement system, which enhances HIV-1 infectivity, HIV-1 actively stimulates the synthesis of complement factor C3 in astrocytes and neurons [310]. This effect may be mediated by the viral proteins gp41 and Nef [310]. C3 deposits and their high activity may play a key role in the pathogenesis of neurological disorders observed in HIV-1 infected individuals. In addition, the binding of anti-HIV antibodies to complement opsonized virions facilitates the interaction of HIV-1 with erythrocytes. HIV-1 binds to red blood cells in a complement system. These HIV-1-associated erythrocytes can not only deliver immunocomplex HIV-1 to organs susceptible to infection, but also free HIV [311]. In this case, the protection of HIV-1 from lysis is due, at least in part, to the presence of complement activation regulators CD59 and CD55 in the viral envelope, which the virus recruits from the host cell during the budding process [312,313,314]. In addition, additional resistance to complement system attacks is provided by the binding of factor H to HIV-1 [315]. Thus, on the one hand, HIV activates the complement system and stimulates the synthesis of complement factors and proinflammatory cytokines. On the other hand, it demonstrates the ability to evade the complement system, using it to enhance its virulence.

Complement system-mediated inflammation modulates thrombotic responses by increasing procoagulant activity, decreasing anticoagulant activity, and suppressing fibrinolysis [316]. Both inflammatory and coagulation biomarkers have been found to be elevated in HIV infection [317,318,319,320,321]. It has been suggested that increased translocation of microbial products across intestinal surfaces, resulting from irreversible damage to mucosal lymphatic tissue, may contribute to monocyte activation, tissue factor expression, and pathogenic hypercoagulability [322,323,324]. It has been reported that in HIV-infected, when compared to uninfected, the level of TF expression in monocytes is higher [325]. TF expression in monocytes correlated with HIV viral load, D-dimer levels, and soluble CD14 (sCD14), a monocyte inflammatory marker and bacterial lipopolysaccharide (LPS) co-receptor [325]. Without prompt treatment, HIV replication leads to increased levels of some procoagulants (eg, factor VIII and von Willebrand factor) due to systemic inflammation, and decreased levels of all major anticoagulants (eg, antithrombin, protein C, and protein S), as well as function-dependent procoagulants hepatocytes. Computer modeling has shown that the net effect of HIV replication is to increase coagulation potential [326]. Thus, it can be assumed that the development of HUS associated with HIV infection may be mediated by activation of the complement system. Moreover, one of the key functions of the complement system in this pathological process is to disrupt the balance between procoagulants and anticoagulants, leading to activation of the blood coagulation system. Nevertheless, these assumptions require careful verification.

### 5.3. Enteroviral Infections

The participation of invasive enteroviral infections in the development of HUS remains questionable today. The first case of HUS associated with enterovirus infection was described back in 1965. Coxsackievirus Group A type 4 was detected in a patient with HUS [327] Subsequently, more than 60 patients with HUS were reported to have enterovirus infections, including Coxsackievirus and ECHO virus [327,328,329,330,331,332,333].The problem is that the diagnoses of enterovirus infection in the cases described were based on serology, obtaining viral cultures from the throat, rectum, and stool, and identifying viral particles in stool samples using electron microscopy. These approaches confirm the very presence of infection, but do not directly link the presence of enterovirus infection with the development of HUS. It should also be noted that patients were not always examined for the presence of hemorrhagic bacterial infection. A comparative study on the presence of enterovirus infections in two groups of patients with HUS, STEC-positive (58 people) and STEC-negative (31 people), did not reveal statistically significant differences between them [329]. The authors of this work proposed to exclude enteroviruses from the list of infectious pathogens that can provoke HUS. However, a number of researchers question this position. Two cases have been described in which enterovirus infection was detected directly in the kidneys of patients with HUS [331,332]. In one case, the viral culture did not give a positive result, but enteroviral RNA was isolated from the kidney tissue. There were no abnormalities in the serological parameters of complement factors, ADAMTS13 activity was normal, pneumococcal infection, *E. coli* serotype O157:H7 infection, shigellosis or salmonellosis were excluded. However, the clinical features and renal biopsy were entirely consistent with HUS [334]. As is the case with some other infections, the mechanisms of pathogenesis of HUS caused by enterovirus infections remain unclear. Most researchers are inclined to believe that they can act as a trigger in people with a genetic predisposition to aHUS. Many serotypes of echovirus (EV) and coxsackievirus B (CBV) have been shown to bind human decay accelerating factor (DAF) and use it as a receptor. [335]. DAF is a glycosylphosphatidylinositol (GPI)-anchored membrane protein that inhibits both the classical and alternative pathways of complement activation, accelerating the dissociation of already formed C3 convertases, and preventing the formation of new ones [336,337,338,339]. The association of viral particles with DAF may be mediated by C3b, and its formation may result in activation of the alternative complement pathway, as has been demonstrated for Coxsackievirus B3 [340]. Another possible way is damage to endothelial cells directly by the virus or as a result of the cytokine storm caused by it. Using Coxsackie B virus as an example, it was shown that viral infection can cause increased expression of adhesion molecules on the surface of endothelial cells and their mediated development of TMA [341]. We were unable to find significant information on the involvement of the blood coagulation system in enterovirus-associated HUS. However, it has been found that during sepsis, enteroviral pathogens cause the release of proinflammatory cytokines and activation of monocytes, which leads to increased regulation of tissue factors. Activation of tissue factors ultimately activate the coagulation cascade. Moreover, cytokines stimulate endothelial cells to produce plasminogen activator inhibitors, thereby reducing fibrinolysis [342]. It cannot be excluded that the development of HUS during enterovirus infections may be mediated by similar mechanisms, when complement system-mediated inflammation leads to activation of the blood coagulation system and the development of TMA.

### 5.4. SARS-CoV-2

The SARS-CoV-2 coronavirus causes an acute, predominantly respiratory infection, which, in especially severe cases, is accompanied by the development of thrombotic microangiopathy and the resulting organ failure. One form of thrombotic microangiopathy that is observed in patients with COVID-19 is HUS. The mechanisms of HUS development during coronavirus infection remain unclear. The data obtained indicate that SARS-CoV-2 infection can provoke the development of HUS independently and is also a potential risk factor for the development of complications in HUS caused by *E. coli* infection [343,344]. Genetic testing of COVID-19 patients with HUS has shown that not all of them are carriers of aHUS-associated mutations [345]. On the one hand, this suggests that the SARS-CoV-2 viral infection may act as a trigger for aHUS. On the other hand, it is possible that the SARS-CoV-2 virus is capable of independently causing HUS, regardless of the presence of genetic abnormalities. There is evidence that infection with the SARS-CoV-2 virus causes complement-mediated inflammation and thrombotic microangiopathy [346]. It has been suggested that the S glycoprotein of the SARS-CoV-2 coronavirus may bind to mannose-binding lectin (MBL) and thereby activate the mannose-associated serine protease MASP2 [347]. In turn, MASP2 activation is the first step of the lectin pathway of complement activation and part of a positive feedback loop leading to sustained activation of the alternative pathway, and the inflammation and initiation of the coagulation cascade [348,349].

Thus, it was found that due to inflammatory reactions in patients with COVID-19, an imbalance of procoagulant and anticoagulant mechanisms occurs, with endothelial dysfunction playing a major role [350,351]. Thus, in patients in the early stages of the disease, the level of fibrinogen increases [352]. However, structural differences in fibrinogen have been found in patients diagnosed with COVID-19, which contribute to clinical differences in thrombotic features associated with COVID-19. These structural differences are, at least in part, mediated by differential sialylation [352]. In addition, platelets from COVID-19 patients were found to have increased levels of P-selectin expression and activity of the MAPK signaling pathway, which mediates the production of thromboxane. Moreover, the level of P-selectin correlated with the level of platelet α-granules marker platelet growth factor PDGF [353]; with this in mind, we can talk about increased activation of the blood coagulation system in patients with COVID-19.

## 6. Conclusions

The list of pathogens of infectious diseases that can cause the development of thrombotic microangiopathies, including HUS, is gradually expanding. The pathogenetic mechanisms of HUS in various infections are very complex and require study in each specific case. HUS can be caused by toxins and enzymes produced by microorganisms, as well as surface antigens of the pathogen. In addition to endothelial cells, platelets, and erythrocytes, other cellular elements of the blood and specialized cells of organs and tissues (lung alveolocytes, kidney podocytes, etc.) may be involved in the pathogenesis of HUS. From the data presented in the literature, it becomes clear that the common link in the pathogenetic mechanisms of HUS in various infectious diseases is the complement system, which is in close interaction with the blood coagulation system. The connection between these systems is bilateral, multilevel, complex, and is ensured by common regulatory mechanisms, which include both complement factors and blood coagulation factors. Normally, the interaction between the complement system and the blood coagulation system provides the body with protection from pathogens. However, we see that, in some cases, a disruption in the functioning of one of the systems can lead to a change in the activity of the other, mediated by general regulatory mechanisms, and, as a consequence, the development of TMA. It is important to remember that one or another infectious agent does not always trigger the development of HUS as a complication of the underlying disease. It appears that the development of HUS due to infection requires a combination of factors that have not yet been fully established. One of these factors, potentially, is the functionally significant mutations in the genes of the complement system proteins. In turn, mutations in these genes by themselves are not sufficient for the development of aHUS. Based on the presented data, we can assume that infectious diseases can act as a trigger for the development of aHUS. At the same time, the close relationship between the complement system and the blood coagulation system and disturbances in their activity in the pathogenesis of HUS suggest that not only mutations in the genes of the complement system factors, but also in the genes of the blood coagulation system factors can act as a risk factor for the development of HUS. This assumption has already been confirmed in some studies [97,100]. The functional role of the complement system and its interaction with the blood coagulation system in the pathogenesis of HUS requires further research.
